# Protective Effects of Gallic Acid on Oxidative and Inflammatory Markers in the Hippocampus and Prefrontal Cortex of a Ketamine-Induced Schizophrenia-like Model

**DOI:** 10.3390/brainsci16070660

**Published:** 2026-06-23

**Authors:** Ali Osman Arslan, Ihsan Cetin, Ozgur Mehmet Yis, Sevdenur Akcay, Guven Akcay

**Affiliations:** 1 Department of Medical Biology, Faculty of Medicine, Bolu Abant İzzet Baysal University, 14030 Bolu, Turkey; aliosmanarslan@ibu.edu.tr; 2 Department of Biochemistry, Faculty of Medicine, Hitit University, 19030 Çorum, Turkey; ihsancetin@hitit.edu.tr; 3 Department of Biochemistry, Faculty of Medicine, Bolu Abant İzzet Baysal University, 14030 Bolu, Turkey; ozgurmehmetyis@ibu.edu.tr; 4 Department of Physiology, Faculty of Medicine, Bolu Abant İzzet Baysal University, 14030 Bolu, Turkey; sevdenuruzunn@gmail.com; 5 Department of Biophysics, Faculty of Medicine, Bolu Abant İzzet Baysal University, 14030 Bolu, Turkey

**Keywords:** gallic acid, ketamine, oxidative stress, neuroinflammation, cytokines

## Abstract

**Highlights:**

**What are the main findings?**

- Gallic Acid significantly improved ketamine-induced schizophrenia-like behavioral deficits, including hyperlocomotion, memory impairment, and depressive-like behavior.
- Gallic Acid reduced pro-inflammatory cytokines (TNF-α, IL-1β, IL-18) and oxidative stress while restoring antioxidant defenses in the hippocampus and prefrontal cortex.

**What are the implications of the main findings?**

- Modulation of neuroinflammation and oxidative stress may represent an effective therapeutic strategy for schizophrenia.
- Gallic Acid shows promise as a neuroprotective adjunct candidate for schizophrenia treatment through its anti-inflammatory and antioxidant properties.

**Abstract:**

Background: Schizophrenia is a chronic neuropsychiatric disorder characterized by cognitive impairment, behavioral abnormalities, neuroinflammation, and oxidative stress. Increasing evidence suggests that dysregulated inflammatory cytokines and impaired antioxidant defenses contribute to schizophrenia pathophysiology. This study investigated the neuroprotective and anti-inflammatory effects of Gallic Acid (GA) in a ketamine-induced experimental schizophrenia model. Methods: Thirty male Balb/C mice were randomly divided into control, ketamine, and ketamine + GA groups. Schizophrenia was induced with ketamine (25 mg/kg/day) for 7 days, while the treatment group additionally received GA (60 mg/kg/day) for another 7 days. Behavioral tests, including open field, novel object recognition, and tail suspension tests, were performed to evaluate locomotor activity, cognition, and depressive-like behavior. Tumor necrosis factor-alpha (TNF-α), interleukin-1 beta (IL-1β), interleukin-18 (IL-18), superoxide dismutase (SOD), catalase (CAT), glutathione peroxidase (GSH-Px), total antioxidant status (TAS), and total oxidant status (TOS) levels were analyzed in hippocampal and prefrontal cortex tissues to assess inflammatory and oxidative stress-related alterations. Results: Ketamine induced schizophrenia-like behaviors, including hyperlocomotion, memory impairment, and increased immobility. These behavioral alterations were accompanied by significantly elevated TNF-α, IL-1β, IL-18, and TOS levels, alongside reduced SOD, CAT, GSH-Px, and TAS levels in the hippocampus and prefrontal cortex. GA treatment ameliorated behavioral impairments, restored antioxidant enzymes, increased TAS levels, and reduced pro-inflammatory cytokines and TOS in these brain regions. Conclusions: GA exerted neuroprotective effects in the ketamine-induced schizophrenia model by reducing oxidative stress, neuroinflammation, and behavioral deficits. These findings suggest that Gallic Acid may serve as a promising therapeutic candidate for schizophrenia through modulation of inflammatory and oxidative stress pathways.

## 1. Introduction

Schizophrenia is a chronic neuropsychiatric disorder affecting approximately 1% of the global population [1]. The disease typically manifests in early adulthood and leads to substantial impairments in social, occupational, cognitive, and emotional functioning [2]. The etiology of schizophrenia is multifactorial, with genetic susceptibility, environmental factors, and neuroinflammatory mechanisms playing critical roles in its development [2,3].

Accumulating evidence indicates that inflammatory processes within the central nervous system and oxidative stress are key contributors to the pathogenesis of schizophrenia [4]. Neuroinflammation is a pivotal process that promotes synaptic plasticity loss, neurodegeneration, and disrupted neural communication in schizophrenia [5,6]. Dysregulation of these mechanisms underlies characteristic cognitive and behavioral symptoms of the disorder, including learning deficits, depressive manifestations, and motor dysfunction. Notably, patients with schizophrenia exhibit significantly elevated levels of inflammatory cytokines and impaired antioxidant defense mechanisms in the hippocampus and prefrontal cortex [7]. Elevated concentrations of pro-inflammatory cytokines such as tumor necrosis factor (TNF)-α, interleukin (IL)-1β, and IL-18 are widely recognized as core biomarkers of neuroinflammatory processes in schizophrenia [8,9,10].

The toxic role of free radicals in schizophrenia pathogenesis was first proposed in the 1950s [11], prompting extensive research into the vulnerability of the brain to oxidative stress due to its high oxygen consumption and relatively weak antioxidant defense systems [12]. Based on these findings, the inflammatory/oxidative hypothesis posits that disruptions in the glutathione system [13] contribute to dopaminergic and glutamatergic signaling abnormalities and exacerbate oxidative stress [14]. Collectively, current evidence underscores the critical involvement of neuroinflammation and impaired antioxidant defenses in schizophrenia, highlighting these pathways as promising therapeutic targets.

Ketamine, particularly at subanesthetic doses, disrupts glutamatergic neurotransmission and induces a robust animal model that mimics the positive, negative, and cognitive symptoms of schizophrenia [15]. This model is widely used to study neuroinflammation and oxidative stress, as ketamine administration has been shown to trigger inflammatory responses and oxidative damage in brain regions such as the hippocampus and prefrontal cortex.

Gallic acid (3,4,5-trihydroxybenzoic acid, GA) is a naturally occurring polyphenolic compound widely found in plants, particularly in tea, grapes, fruits, nuts, and various medicinal herbs [16]. Despite its relatively low molecular weight (170.12 g/mol) and hydrophilic nature, its bioavailability can be enhanced through conjugation with lipophilic moieties or ester derivatives [17,18]. Gallic acid exhibits potent antioxidant, anti-inflammatory, and neuroprotective properties by neutralizing reactive oxygen species, reducing lipid peroxidation, and supporting endogenous cellular defense mechanisms [19,20]. Furthermore, several studies have demonstrated that gallic acid is capable of crossing the blood–brain barrier and modulating both oxidative stress and microglial activation within the central nervous system [21]. Indeed, gallic acid has been shown to reduce amyloid-beta accumulation in Alzheimer’s disease, protect dopaminergic neurons against oxidative stress in Parkinson’s disease, and suppress neuroinflammation following traumatic brain injury, thereby exhibiting a consistent neuroprotective profile [22,23,24,25,26]. These pleiotropic effects position gallic acid as a promising natural compound for the treatment of neuropsychiatric disorders.

In the pathophysiology of schizophrenia, oxidative stress and neuroinflammation are considered two interdependent pathological processes that perpetuate one another. In this context, TNF-α, IL-1β, and IL-18 are regarded as key inflammatory biomarkers. Excessive production of TNF-α and IL-1β enhances microglial activation, creating a neurotoxic environment and contributing to neuronal dysfunction [27]. IL-18, in particular, not only amplifies inflammatory responses but may also disrupt dopaminergic and glutamatergic balance, thereby contributing to psychotic symptoms. Elevated levels of these cytokines further exacerbate oxidative stress, establishing a self-perpetuating pathological cycle.

At the cellular level, the most reliable indicators of oxidative stress are the activities of antioxidant defense enzymes, including superoxide dismutase (SOD), catalase (CAT), and glutathione peroxidase (GSH-Px). SOD constitutes the first line of defense by converting superoxide anion radicals into hydrogen peroxide, which is subsequently detoxified into water and oxygen by CAT and GSH-Px [28]. In pathological conditions such as schizophrenia, suppression of these enzymatic activities leads to excessive accumulation of reactive oxygen species, resulting in lipid, protein, and DNA damage, ultimately culminating in neuronal cell death and synaptic dysfunction [29].

Gallic acid exerts its therapeutic potential precisely along these two pathological axes. On one hand, it reduces neuroinflammation by inhibiting the synthesis and release of pro-inflammatory cytokines such as TNF-α, IL-1β, and IL-18 [30]. On the other hand, owing to its phenolic structure, gallic acid acts as a direct free radical scavenger and simultaneously enhances endogenous antioxidant capacity by increasing the activities of SOD, CAT, and GSH-Px [31]. Through these mechanisms, gallic acid may interrupt the vicious cycle between oxidative stress and neuroinflammation in schizophrenia, thereby conferring neuroprotective effects.

The present study aimed to investigate the neuroprotective and anti-neuroinflammatory effects of gallic acid in a ketamine-induced schizophrenia model. The central hypothesis was that gallic acid treatment would attenuate ketamine-induced neuroinflammation and oxidative stress-related neuronal damage. Accordingly, levels of TNF-α, IL-1β, and IL-18, as well as the activities of SOD, CAT, and GSH-Px, were examined in the hippocampus and prefrontal cortex, alongside an evaluation of behavioral alterations in the animal model.

The findings demonstrated a significant increase in inflammatory cytokine levels and a marked reduction in antioxidant enzyme activities in the ketamine-induced schizophrenia model. Following gallic acid treatment, a statistically significant decrease in inflammatory markers and a concomitant increase in antioxidant enzyme activities were observed. Moreover, improvements were detected in behavioral test outcomes indicative of neuronal recovery. Collectively, these results suggest that gallic acid may exert beneficial effects in this experimental model and warrant further investigation as a potential therapeutic agent for schizophrenia.

## 2. Materials and Methods

**Figure 1:**
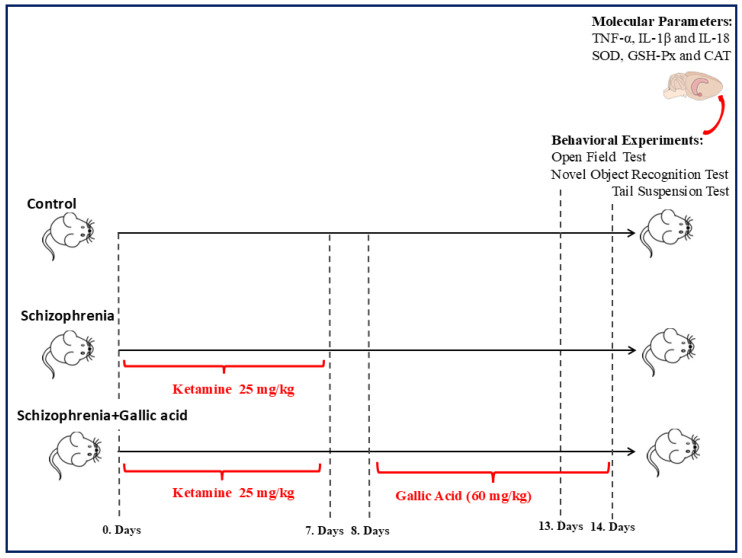
Timeline of the experimental procedure.

Two-month-old male Balb/C mice weighing 25–30 g were obtained from the Experimental Animals Application. Ten animals were housed per cage with ad libitum access to food and water. Mice were maintained at room temperature (22–26 °C) under a 12 h light/dark cycle (lights on from 7:00 a.m. to 7:00 p.m.). Mice were divided into three groups: Control (n = 10), Ketamine-Induced Schizophrenia-Like Group (Ketamine Group, n = 10), and Ketamine + Gallic Acid Group (n = 10). Animals were randomly assigned to the groups. The Control group received 0.9% NaCl. The Ketamine-Induced Schizophrenia-Like Group (hereafter referred to as the Ketamine Group) was administered ketamine (25 mg/kg/day; Ketasol 10%, Richter Pharma AG, Wels, Austria) for seven consecutive days to induce schizophrenia-like behavioral and neurochemical alterations, as previously described [32,33]. The Ketamine + Gallic Acid Group received ketamine (25 mg/kg/day) for seven days, followed by gallic acid (60 mg/kg/day) for the subsequent seven days [34]. The dose of gallic acid (60 mg/kg/day) was selected based on previous studies demonstrating antioxidant and neuroprotective effects without evidence of toxicity. Intraperitoneal administration was preferred because it provides reliable systemic exposure and has been widely used in previous experimental studies evaluating the neuroprotective effects of gallic acid (Figure 1). All animal procedures were approved by the Erciyes University Local Ethics Committee for Animal Experiments (Approval No. 22/133) and conducted in accordance with ARRIVE 2.0 guidelines.

### 2.1. Behavioral Experiments

#### 2.1.1. Open Field Test

The Open Field Test is a widely used behavioral test to evaluate locomotor activity and anxiety levels in experimental animals. In this study, the apparatus consisted of a black square arena with a base area of 40 × 40 cm and a height of 40 cm. The floor was divided into 16 equal squares. According to the experimental protocol, each mouse was individually placed in the center of the arena and observed for 3 min. Total distance and velocity were recorded by the video camera [35,36].

#### 2.1.2. Novel Object Recognition Test

The novel object recognition test is an effective method for evaluating attention and short-term memory and consists of habituation, training, and memory phases. During the habituation phase, mice were placed in the apparatus (40 × 40 × 40 cm) and allowed to freely explore the empty environment for 5 min. In the training phase, two objects were placed in the apparatus, and mice were allowed to explore these objects for 5 min. After each trial, the apparatus was cleaned with 70% ethanol and allowed to dry to prevent odor-related effects. In the memory phase, one of the objects was replaced with a novel object, and the behavior of the mice was recorded for 5 min. Mice are expected to spend a longer time exploring the novel object [37,38]. In the novel object recognition test, the discrimination index and the time spent on the novel object (seconds) were analyzed. The discrimination index was calculated using the following formula:

Discrimination Index = ((Time spent on novel object − Time spent on familiar object)/Total exploration time) × 100

#### 2.1.3. Tail Suspension Test

In the tail suspension test, each mouse was suspended at a height of 30 cm from the ground using adhesive tape placed approximately 1 cm from the tip of the tail. During the last 4 min of the 6 min test period, the total duration of immobility—defined as motionless hanging without any effort—was recorded. Mice that climbed their tails during the test were excluded from the experiment [39].

### 2.2. Tissue Preparation

Following behavioral testing, mice were deeply anesthetized with ketamine/xylazine and euthanized by cervical dislocation. The brains were rapidly removed, and the hippocampus and prefrontal cortex were carefully dissected on ice. Tissue samples were homogenized (1:10, *w*/*v*) in ice-cold phosphate-buffered saline (PBS, pH 7.4) using a tissue homogenizer. The homogenates were centrifuged at 10,000× *g* for 15 min at 4 °C, and the resulting supernatants were collected for biochemical analyses. Total protein concentrations were determined using the Bradford method with bovine serum albumin (BSA) as the standard. The supernatants were aliquoted and stored at −80 °C until further analysis.

### 2.3. Measurement of TNF-α, IL-1β, and IL-18 Levels

The levels of TNF-α, IL-1β, and IL-18 were determined using commercially available mouse-specific sandwich ELISA kits (Mouse TNF-α ELISA Kit, Cat. No. E-EL-M0049; Mouse IL-1β ELISA Kit, Cat. No. E-EL-M0037; Mouse IL-18 ELISA Kit, Cat. No. E-EL-M0730; Elabscience Biotechnology Co., Ltd., Wuhan, China) according to the manufacturer’s instructions.

Briefly, standards and tissue supernatants were added to microplates pre-coated with monoclonal antibodies specific for each cytokine and incubated at 37 °C for 90 min. After washing, biotinylated detection antibodies were added and incubated for 60 min, followed by incubation with horseradish peroxidase (HRP)-conjugated streptavidin for 30 min. Subsequently, tetramethylbenzidine (TMB) substrate solution was added, and color development was allowed to proceed in the dark. The reaction was terminated using a stop solution, and absorbance was measured at 450 nm using a microplate reader. Cytokine concentrations were calculated from standard calibration curves generated using recombinant cytokine standards supplied with the kits and were expressed as pg/mL.

### 2.4. Determination of Antioxidant Enzyme Activities

#### 2.4.1. Superoxide Dismutase (SOD) Activity

SOD activity was measured according to the method described by Sun et al. Briefly, superoxide radicals generated by the xanthine–xanthine oxidase system reduce 2-(4-iodophenyl)-3-(4-nitrophenol)-5-phenyltetrazolium chloride (INT) to form a red-colored formazan dye. SOD inhibits this reaction by scavenging superoxide radicals. The absorbance of the resulting chromogen was measured at 505 nm, and enzyme activity was expressed as U/mg protein [40].

#### 2.4.2. Glutathione Peroxidase (GSH-Px) Activity

GSH-Px activity was determined according to the method of Paglia and Valentine. In this coupled enzymatic assay, glutathione peroxidase catalyzes the reduction of hydrogen peroxide by reduced glutathione (GSH), while glutathione reductase regenerates GSH using NADPH. The oxidation of NADPH to NADP^+^ was monitored spectrophotometrically at 340 nm. Results were expressed as U/mg protein.

#### 2.4.3. Catalase (CAT) Activity

Catalase activity was determined using a commercial colorimetric assay kit (Elabscience Biotechnology Co., Ltd., Wuhan, China) according to the manufacturer’s protocol. Catalase decomposes hydrogen peroxide (H_2_O_2_) into water and oxygen. Residual H_2_O_2_ reacts with the chromogenic reagents in the assay mixture, producing a colored compound that was measured at 570 nm using a microplate reader. Results were expressed as U/mg protein [41].

### 2.5. Determination of Total Antioxidant Status (TAS) and Total Oxidant Status (TOS)

Total antioxidant status (TAS) and total oxidant status (TOS) levels were determined using commercial colorimetric assay kits (Elabscience Biotechnology Co., Ltd., Wuhan, China) according to the manufacturer’s instructions.

TAS is an operational measure of the cumulative antioxidant capacity of the antioxidants detectable under the assay conditions and is expressed as mmol Trolox equivalent/L. Rather than measuring individual antioxidant molecules, the assay estimates the combined reducing capacity of antioxidant constituents present in the tissue homogenate. Therefore, TAS reflects the overall antioxidant capacity measurable by this specific analytical method rather than the absolute total antioxidant activity of the biological sample.

TOS represents the cumulative oxidant burden detectable by the assay and is expressed as μmol H_2_O_2_ equivalent/L. The assay estimates the total concentration of oxidizing substances present in the sample under the defined reaction conditions and provides an index of oxidative status rather than a direct measurement of all oxidant species.

Briefly, tissue supernatants were added to microplate wells containing the manufacturer-provided assay reagents. Following incubation under the recommended conditions, absorbance values were measured using a microplate reader at the wavelengths specified by the manufacturer. TAS concentrations were calculated from calibration curves generated using Trolox standards, whereas TOS concentrations were calculated using hydrogen peroxide standards. All samples were analyzed in duplicate [42,43].

Since TAS and TOS measurements are method-dependent and influenced by assay chemistry and reaction conditions, the obtained values should be interpreted as indices of antioxidant capacity and oxidant burden detectable by the employed assay system rather than comprehensive measurements of all antioxidant, antiradical, or oxidant activities present in the tissue samples.

### 2.6. Statistical Analysis

Statistical analyses were performed using SPSS software (version 20.0; IBM Corp., Armonk, NY, USA). Data distribution was assessed using the Shapiro–Wilk test. For normally distributed data, one-way analysis of variance (ANOVA) followed by Tukey’s post hoc test was applied for multiple group comparisons. For non-normally distributed data, the Kruskal–Wallis test was used, followed by Dunn’s or Bonferroni-adjusted pairwise comparisons. All data are presented as mean ± standard error of the mean (SEM). A *p*-value of <0.05 was considered statistically significant.

## 3. Results

This study evaluated the neurotherapeutic and anti-inflammatory effects of gallic acid in an experimental ketamine-induced schizophrenia model. The findings obtained indicate that gallic acid administration was associated with reduced inflammatory responses, attenuated oxidative stress-related damage, and improved neurological functions in this model.

**Figure 2:**
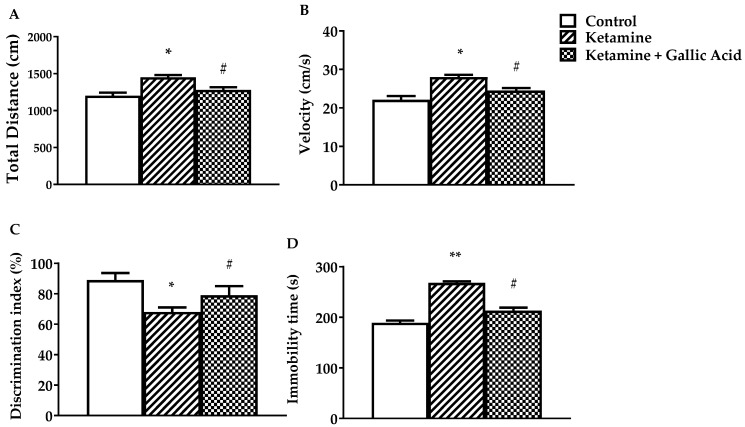
Behavioral test results of the experimental groups. (**A**) Total distance in OF (cm), (**B**) velocity in OF (cm/s), (**C**) discrimination index in NOR (%), (**D**) immobility time (s) (n = 10 for each group; * *p* < 0.05, ** *p* < 0.001 indicate difference compared to the control group, and # *p* < 0.05 indicates difference compared to the ketamine group; one-way ANOVA, post hoc Tukey test). All data are presented as mean ± S.E.M.

In the ketamine group, a significant increase in locomotor activity was observed compared to the control group, whereas a significant decrease in this activity was detected in the ketamine + gallic acid group following gallic acid treatment (Figure 2). Similarly, an improvement trend was observed in learning and memory tests as well as in the depression test. In the ketamine group, the discrimination index in the novel object recognition (NOR) test decreased, while immobility time increased in the depression test. In contrast, in the group receiving gallic acid treatment, the discrimination index increased, and immobility time decreased (Figure 2).

**Figure 3:**
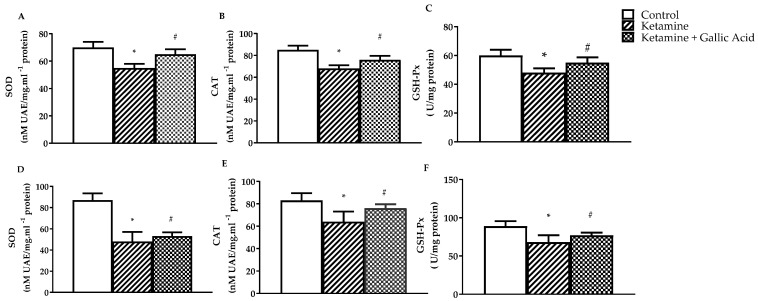
Oxidative stress results. (**A**) SOD level in hippocampus, (**B**) CAT level in hippocampus, (**C**) GSH-Px activity in hippocampus, (**D**) SOD level in prefrontal cortex, (**E**) CAT level in prefrontal cortex, (**F**) GSH-Px activity in prefrontal cortex (n = 10 for each group; * *p* < 0.05 indicates difference compared to the control group, and # *p* < 0.05 indicates difference compared to the ketamine group; one-way ANOVA, post hoc Tukey test). All data are presented as mean ± S.E.M.

In the hippocampus and prefrontal cortex tissues, a significant decrease in SOD, CAT, and GSH-Px activities was observed in the ketamine group compared to the control group (*p* < 0.05). Following gallic acid treatment, a significant increase in SOD, CAT, and GSH-Px activities was detected in the hippocampus and prefrontal cortex tissues of the ketamine + gallic acid group compared to the ketamine group (*p* < 0.05) (Figure 3).

**Figure 4:**
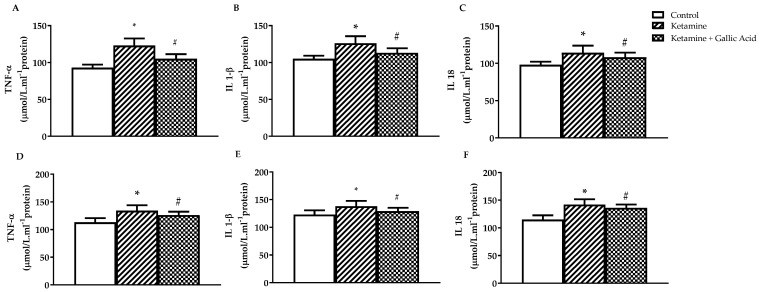
Inflammation results. (**A**) TNF-α level in hippocampus, (**B**) IL-1β level in hippocampus, (**C**) IL-18 level in hippocampus, (**D**) TNF-α level in prefrontal cortex, (**E**) IL-1β level in prefrontal cortex, (**F**) IL-18 level in prefrontal cortex (n = 10 for each group; * *p* < 0.05 indicates difference compared to the control group, and # *p* < 0.05 indicates difference compared to the ketamine group; one-way ANOVA, post hoc Tukey test). All data are presented as mean ± S.E.M.

In the hippocampus and prefrontal cortex tissues, a significant increase in TNF-α, IL-1β, and IL-18 levels was observed in the ketamine group compared to the control group (*p* < 0.05) (Figure 4). Following gallic acid treatment, a significant decrease in TNF-α, IL-1β, and IL-18 levels was detected in the hippocampus and prefrontal cortex tissues of the ketamine + gallic acid group compared to the ketamine group (*p* < 0.05) (Figure 4).

**Figure 5:**
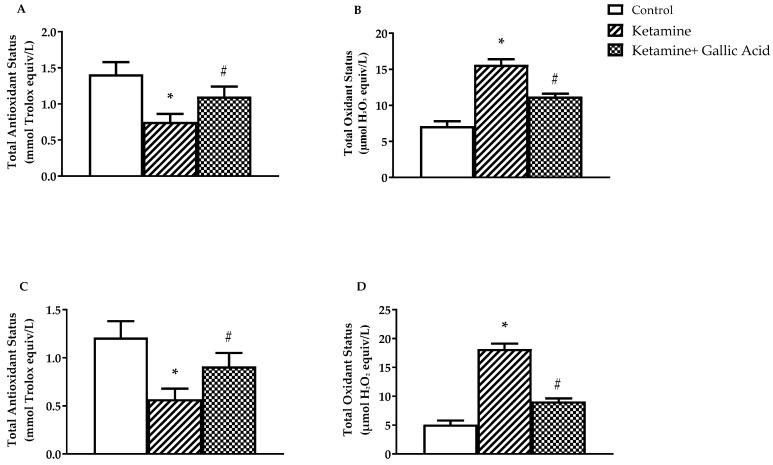
Effects of gallic acid on oxidative stress parameters in the ketamine-induced schizophrenia model. (**A**) Hippocampal TAS, (**B**) Hippocampal TOS, (**C**) Prefrontal cortex TAS, and (**D**) Prefrontal cortex TOS levels. Data are presented as mean ± SEM. * *p* < 0.05 vs. control group; # *p* < 0.05 vs. ketamine group.

In addition to inflammatory parameters, TAS and TOS levels were also evaluated in the hippocampus and prefrontal cortex tissues. In the hippocampal tissue, TAS levels were significantly decreased in the ketamine group compared to the control group (*p* < 0.05). In contrast, gallic acid treatment significantly increased TAS levels compared to the ketamine group (*p* < 0.05) (Figure 5A). Similarly, hippocampal TOS levels were significantly elevated in the ketamine group compared to the control group (*p* < 0.05), whereas gallic acid administration significantly reduced TOS levels (*p* < 0.05) (Figure 5B).

Similar findings were observed in the prefrontal cortex tissue. TAS levels were significantly reduced in the ketamine group compared to the control group (*p* < 0.05), while gallic acid treatment significantly increased TAS levels (*p* < 0.05) (Figure 5C). Conversely, TOS levels in the prefrontal cortex were significantly increased in the ketamine group (*p* < 0.05), and gallic acid treatment significantly suppressed this increase (*p* < 0.05) (Figure 5D). These findings further support that ketamine administration increased oxidative stress in both the hippocampus and prefrontal cortex, whereas gallic acid treatment attenuated oxidative damage by enhancing the antioxidant defense system.

## 4. Discussion

In this study, the potential anti-inflammatory and antioxidant effects of gallic acid were investigated in a ketamine-induced schizophrenia-like experimental model. Ketamine is widely used as a pharmacological tool to study schizophrenia-related behavioral and biochemical alterations by inducing glutamatergic dysfunction through NMDA receptor antagonism [44]. In particular, high-dose or repeated administration protocols have been reported to reproduce schizophrenia-like phenotypes such as impaired synaptic plasticity, neuroinflammatory responses, and cognitive deficits [45]. However, it should be noted that ketamine may also exert antidepressant-like effects at low doses and under different administration regimens, mediated by distinct neurobiological mechanisms [46]. Therefore, interpretation of findings obtained from this model requires careful consideration of its dose-dependent and bidirectional pharmacological properties.

Ketamine induces glutamatergic dysfunction via NMDA receptor antagonism and has been associated with the activation of oxidative stress-related pathways. Hou et al. demonstrated that ketamine induces significant neuronal damage, particularly in the hippocampus and prefrontal cortex, by disrupting antioxidant defense systems and compromising neuronal integrity. Consistently, the increased TOS and decreased TAS levels observed in the present study align with these findings [47].

In the present study, ketamine administration was associated with behavioral alterations in mice, including increased locomotor activity, impaired novel object recognition performance, and prolonged immobility time in the tail suspension test. These behavioral changes are consistent with previously reported ketamine-induced schizophrenia-like phenotypes [12]. Alongside these behavioral alterations, elevated levels of pro-inflammatory cytokines—TNF-α, IL-1β, and IL-18—were detected in both the hippocampus and prefrontal cortex. These findings suggest the presence of a neuroinflammatory response following ketamine-induced glutamatergic dysregulation.

TNF-α has been reported to increase blood–brain barrier permeability, potentially facilitating peripheral immune cell infiltration and contributing to neuroinflammatory processes [48]. Notably, the concurrent elevation of IL-1β and IL-18 levels with impaired cognitive performance in the novel object recognition test raises the possibility that these cytokines may be involved in cognitive deficits, as previous studies have suggested their inhibitory effects on synaptic plasticity and long-term potentiation (LTP) [49].

The elevation in pro-inflammatory cytokines observed in the ketamine-treated group may be related to microglial activation and downstream signaling pathways, particularly nuclear factor kappa-B (NF-κB), which is a key regulator of inflammatory mediator expression. Additionally, the reduced activities of antioxidant enzymes (SOD, CAT, and GSH-Px) indicate increased oxidative stress, which may further activate redox-sensitive transcriptional pathways and contribute to sustained inflammatory responses. The interplay between oxidative stress and neuroinflammation is considered a key contributor to neuronal dysfunction and behavioral impairments in schizophrenia-like conditions.

Oxidative stress has been widely implicated in schizophrenia-related pathophysiology. Specifically, excessive production of reactive oxygen species in brain regions involved in cognition, such as the hippocampus and prefrontal cortex, may lead to lipid peroxidation, protein oxidation, and neuronal dysfunction. Murray et al. reported that oxidative stress represents a core biological mechanism in schizophrenia spectrum disorders and that impairment of antioxidant defenses correlates with disease severity. Consistently, the decreased TAS levels and increased TOS levels observed in the ketamine-treated groups strongly support this notion [29].

In the present study, oxidative stress parameters were evaluated in the hippocampus and prefrontal cortex using a ketamine-induced schizophrenia-like model, and the potential protective effects of gallic acid were investigated. Gallic acid treatment was associated with increased TAS levels and decreased TOS levels in both brain regions. These findings suggest that gallic acid may attenuate ketamine-induced oxidative imbalance and restore redox homeostasis.

Treatment with gallic acid was associated with improvements in both behavioral and biochemical parameters in the ketamine-induced schizophrenia-like model. Behaviorally, gallic acid-treated animals exhibited reduced locomotor activity, improved novel object recognition performance, and decreased immobility time, suggesting a potential beneficial effect on ketamine-induced behavioral alterations. Biochemically, gallic acid administration was accompanied by increased activities of antioxidant enzymes (SOD, GSH-Px, and CAT) and reduced levels of pro-inflammatory cytokines (TNF-α, IL-1β, and IL-18) in the hippocampus and prefrontal cortex.

The anti-inflammatory effects observed following gallic acid treatment may involve multiple interconnected mechanisms. One possible explanation is the modulation of the NF-κB signaling pathway, which may be indirectly influenced by the antioxidant properties of gallic acid. Since reactive oxygen species (ROS) can act as secondary messengers in NF-κB activation, the free radical scavenging capacity of gallic acid might contribute to the attenuation of this pathway. However, this interpretation remains speculative, as NF-κB activity was not directly assessed in the present study.

**Figure 6:**
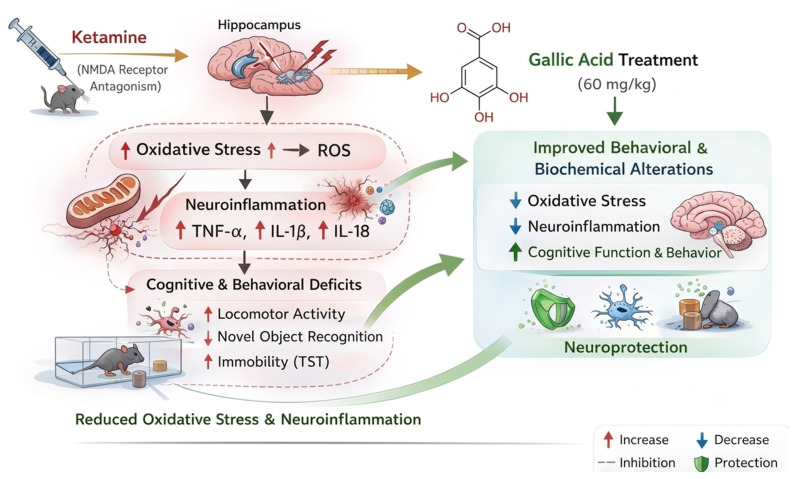
Simplified schematic illustrating ketamine-induced oxidative stress and neuroinflammation leading to behavioral deficits and the therapeutic effects of gallic acid through antioxidant and anti-inflammatory mechanisms.

Furthermore, the increase in antioxidant enzyme activities suggests that gallic acid may support endogenous antioxidant defense systems, potentially reducing oxidative stress and its downstream inflammatory consequences. The restoration of SOD, CAT, and GSH-Px activities indicates that gallic acid may contribute both to the neutralization of reactive oxygen species and to the enhancement of cellular antioxidant capacity (Figure 6).

Similarly, Ben-Azu et al. reported that diosgenin ameliorated oxidative stress and strengthened antioxidant defenses in ketamine-induced schizophrenia models. Their study demonstrated that ketamine suppressed GSH, SOD, and CAT activities while increasing oxidative damage markers. In agreement with these findings, the present study showed that gallic acid increased TAS levels and decreased TOS levels, suggesting that it may exert antioxidant effects in a similar experimental context [50].

The relationship between oxidative stress and neuroinflammation is complex and bidirectional; oxidative stress can trigger inflammatory responses, while inflammation may further enhance oxidative damage. By modulating both processes, gallic acid appears to interfere with this pathological cycle. In line with this, Rume et al. reported that eugenol reduced ketamine-induced schizophrenia-like behavioral alterations, neuroinflammation, and oxidative stress, suggesting that phenolic compounds may attenuate ketamine-associated neurotoxicity [51]. Similarly, our findings suggest that gallic acid may exert comparable beneficial effects on oxidative stress parameters.

Furthermore, the reduction in IL-1β and IL-18 levels observed in the present study is noteworthy, as these cytokines have been implicated in synaptic dysfunction and cognitive impairment [49]. For instance, IL-1β has been reported to impair long-term potentiation and memory formation, while IL-18 may influence neurotransmitter regulation. Therefore, the reduction in these cytokines following gallic acid treatment may be associated with the observed improvements in cognitive performance.

Additionally, the ability of gallic acid to cross the blood–brain barrier and exert effects within the central nervous system has been previously reported [21]. This property may allow gallic acid to influence neuroinflammatory processes at the cellular level [52]. Microglia, when activated, can produce pro-inflammatory cytokines and reactive oxygen species, which may contribute to neuronal dysfunction. It is possible that gallic acid may modulate microglial activation, thereby supporting neuronal and synaptic function; however, this mechanism remains speculative in the absence of direct experimental evidence.

The behavioral changes observed in this study—including reduced hyperlocomotion, improved novel object recognition performance, and decreased immobility time—are consistent with the biochemical alterations. Reduced hyperlocomotion in ketamine-treated animals following gallic acid administration may reflect attenuation of ketamine-induced behavioral dysregulation rather than direct antipsychotic-like effects. Improvement in novel object recognition performance may indicate enhanced cognitive processing, which is relevant in models of cognitive impairment. Similarly, reduced immobility time in the tail suspension test may suggest modulation of affective-like behaviors; however, interpretation should be made cautiously given the limitations of the model.

Several limitations of this study should be acknowledged. First, the use of only male mice limits the generalizability of the findings, as potential sex-dependent differences could not be evaluated; estrogen-related anti-inflammatory mechanisms may influence the response to gallic acid. Second, key mechanistic pathways such as NF-κB signaling, Nrf2/HO-1 axis, MAPK pathway, and NLRP3 inflammasome activation were not directly assessed, which limits mechanistic interpretation. Third, although the ketamine model reproduces certain schizophrenia-like behavioral and biochemical alterations, it does not fully reflect the chronic and developmental nature of schizophrenia. Additionally, the use of a single dose of gallic acid does not allow evaluation of dose–response relationships, and pharmacokinetic data such as brain or plasma concentrations were not measured. Therefore, future studies incorporating both sexes, multiple dosing regimens, mechanistic pathway analyses, and histological validation would provide more comprehensive insights.

## 5. Conclusions

In conclusion, gallic acid was associated with improvements in ketamine-induced schizophrenia-like behavioral and biochemical alterations in mice. These effects were characterized by attenuation of oxidative stress, reduction in pro-inflammatory cytokine levels, and enhancement of endogenous antioxidant defense systems, as reflected by improved TAS/TOS balance and restored antioxidant enzyme activities.

The consistency between behavioral and biochemical findings suggests a potential beneficial role of gallic acid in modulating oxidative stress- and inflammation-related alterations in this experimental model. However, due to the absence of direct mechanistic, pharmacokinetic, and histopathological evidence, these findings should be interpreted cautiously.

Overall, gallic acid may represent a promising natural compound for further investigation in preclinical models of neuroinflammation and oxidative stress-related neuropsychiatric conditions. Further studies are required to elucidate its molecular mechanisms, optimal dosing strategies, and translational relevance.

## Data Availability

All data supporting the findings of this study are included within the manuscript. Additional datasets are available from the corresponding author upon reasonable request.

## Abbreviations

The following abbreviations are used in this manuscript:

ANOVA	Analysis of Variance
BBB	Blood–Brain Barrier
CAT	Catalase
ELISA	Enzyme-Linked Immunosorbent Assay
GA	Gallic Acid
GSH-Px	Glutathione Peroxidase
H_2_O_2_	Hydrogen Peroxide
HRP	Horseradish Peroxidase
IL	Interleukin
IL-1β	Interleukin-1 Beta
IL-18	Interleukin-18
INT	Iodonitrotetrazolium Chloride
i.p.	Intraperitoneal
LTP	Long-Term Potentiation
NADP^+^	Nicotinamide Adenine Dinucleotide Phosphate
NADPH	Reduced Nicotinamide Adenine Dinucleotide Phosphate
NF-κB	Nuclear Factor Kappa-B
NLRP3	NOD-Like Receptor Family Pyrin Domain Containing 3
NMDA	N-Methyl-D-Aspartate
NOR	Novel Object Recognition
OF	Open Field
ROS	Reactive Oxygen Species
SEM	Standard Error of the Mean
SOD	Superoxide Dismutase
SPSS	Statistical Package for the Social Sciences
TNF-α	Tumor Necrosis Factor-Alpha
